# Differentiation between multiple sclerosis and neuromyelitis optica spectrum disorder using a deep learning model

**DOI:** 10.1038/s41598-023-38271-x

**Published:** 2023-07-19

**Authors:** Jin Myoung Seok, Wanzee Cho, Yeon Hak Chung, Hyunjin Ju, Sung Tae Kim, Joon-Kyung Seong, Ju-Hong Min

**Affiliations:** 1 Department of Neurology, Soonchunhyang University Hospital Cheonan, Soonchunhyang University College of Medicine, Cheonan, South Korea; 2 Department of Artificial Intelligence, Korea University, Seoul, South Korea; 3 Department of Neurology, Samsung Medical Center, Sungkyunkwan University School of Medicine, Seoul, South Korea; 4 Department of Neurology, Neuroscience Center, Samsung Medical Center, Seoul, South Korea; 5 Department of Radiology, Samsung Medical Center, Sungkyunkwan University School of Medicine, Seoul, South Korea; 6 School of Biomedical Engineering, Korea University, Seoul, South Korea; 7 Interdisciplinary Program in Precision Public Health, Korea University, Seoul, South Korea; 8 Department of Health Sciences and Technology, Samsung Advanced Institute for Health Sciences & Technology (SAIHST), Sungkyunkwan University, 50 Irwon-dong, Gangnam-gu, Seoul, 135-710 South Korea

**Keywords:** Neuroscience, Neurology, Machine learning

## Abstract

Multiple sclerosis (MS) and neuromyelitis optica spectrum disorder (NMOSD) are autoimmune inflammatory disorders of the central nervous system (CNS) with similar characteristics. The differential diagnosis between MS and NMOSD is critical for initiating early effective therapy. In this study, we developed a deep learning model to differentiate between multiple sclerosis (MS) and neuromyelitis optica spectrum disorder (NMOSD) using brain magnetic resonance imaging (MRI) data. The model was based on a modified ResNet18 convolution neural network trained with 5-channel images created by selecting five 2D slices of 3D FLAIR images. The accuracy of the model was 76.1%, with a sensitivity of 77.3% and a specificity of 74.8%. Positive and negative predictive values were 76.9% and 78.6%, respectively, with an area under the curve of 0.85. Application of Grad-CAM to the model revealed that white matter lesions were the major classifier. This compact model may aid in the differential diagnosis of MS and NMOSD in clinical practice.

## Introduction

Multiple sclerosis (MS) and neuromyelitis optica spectrum disorder (NMOSD) are autoimmune inflammatory disorders of the central nervous system (CNS) that have similar clinical features^1,2^. A disease-specific autoantibody targeting aquaporin-4 (AQP4 antibody) has been discovered in NMOSD, which can help differentiate NMOSD from MS^3^. However, the antibody assay has variable sensitivity^4^ and can produce false-negative results^5,6^; in addition, levels of antibody could decrease during NMOSD remission^7,8^. In terms of brain magnetic resonance imaging (MRI) lesions in MS and NMOSD, the presence of a lesion in the inferior temporal lobe or adjacent to the lateral ventricle, a U-fiber lesion, or a Dawson’s finger-type lesion are more suggestive of MS than NMOSD. In contrast, longitudinally extensive transverse myelitis, extensive hemispheric lesions, and periependymal lesions are observed mainly in NMOSD^9,10^. Nevertheless, differentiation between MS and NMOSD can still be challenging in specific clinical situations^11^.

Recent machine-learning algorithms have been applied clinically in various neurological diseases^12^. In CNS demyelinating disorders, various aspects of the diseases have been evaluated with machine-learning methods. Some authors have reported promising results applying machine learning methods to user-defined features including clinical characteristics, T2 lesion volume, regional gray matter volume, and regional fractional anisotropy values to differentiate NMOSD from MS^13^. However, only a few studies have applied deep learning algorithms to differentiate NMOSD from MS^14^.

In this study, we aimed to develop a compact and robust deep learning model to differentiate MS and NMOSD using brain MRI data and offer visual explanations for the resulting classification.

## Results

### Demographic and clinical features

Eighty-six patients with MS and 70 patients with NMOSD were finally enrolled in this study; 199 MRI scans (86 baseline and 113 follow-up scans) from patients with MS and 109 MRI scans (70 baseline and 39 follow-up scans) from patients with NMOSD were used for classification modeling (Table 1). MS patients were younger than patients with NMOSD (MS, 35.0 ± 9.9 years; NMOSD, 43.9 ± 12.6 years; *P* < 0.001); at the time MRI scan, most of the MS patients (92.5%) were relapsing–remitting type MS (RRMS). Proportions of females were not significantly different between the two groups (MS, 72.1%; NMOSD, 85.7%; *P* = 0.063). All patients were seronegative for the myelin oligodendrocyte glycoprotein autoantibody (MOG antibody), and most patients with NMOSD (66 of 70, 94.3%) were seropositive for the AQP4 antibody. The neurologic disability at the time of the MRI scans in patients with NMOSD or MS were different; the NMOSD group demonstrated a higher EDSS score compared to that of the MS group (median EDSS score, 2.5 vs. 1.0, respectively; P < 0.001).

**Table 1:** Demographic characteristics of enrolled patients with multiple sclerosis and neuromyelitis optica spectrum disorder.

	Enrolled patients with MS N = 86	Enrolled patients with NMOSD N = 70	P value
Age, years (SD)	35.0 (9.9)	43.9 (12.6)	< 0.001
Female, n (%)	62 (72.1)	60 (85.7)	0.063
AQP4 antibody, n (%)^a^	0 (0)	66 (94.3)	< 0.001
Total number of MRI scans	199	109	< 0.001
Baseline scans, n (%)	86 (43.2)	70 (64.2)	
Follow-up scans, n (%)	113 (56.8)	39 (35.8)	
Disease duration at the time of MRI, years (SD)	5.8 (5.3)	4.8 (5.4)	0.020
EDSS at the time of MRI, median (IQR)	1.00 (0–2.5)	2.5 (1.5–4.0)	< 0.001
Clinical status at the time of MRI			0.106
In acute relapse, n (%)	44 (22.1)	34 (31.2)	
In remission, n (%)	155 (77.9)	75 (68.8)	
Characteristics of brain MRI in NMOSD^b^			
Normal MRI	N/A	21 (19.3)	
Non-specific brain lesions	N/A	38 (34.9)	
Longitudinal corticospinal tracts lesion, n (%)	N/A	11 (10.1)	
Extensive hemispheric lesion, n (%)	N/A	16 (14.7)	
Periependymal lesion, n (%)	N/A	42 (38.5)	
Cervicomedullary lesion, n (%)	N/A	6 (5.5)	
Barkhof criteria fulfillment, n (%)	164 (82.4)	N/A	
Presence of T1 enhancing MS lesion, n (%)^c^	38/198 (19.2)	N/A	

MS, Multiple sclerosis; NMOSD, Neuromyelitis optica spectrum disorder; SD, Standard deviation; AQP4, Aquaporin-4; MRI, Magnetic resonance imaging; IQR, inter-quartile range; N/A, not applicable.

^a^MOG antibody was negative for all enrolled patients.

^b^A single MRI scan can have multiple findings.

^c^One MRI scan was taken without enhancement.

### Conventional MRI findings

Mean disease duration at the time of MRI was 5.4 ± 5.4 years, which differed between the two diseases (MS, 5.8 ± 5.3 years; NMOSD, 4.8 ± 5.4 years; *P* = 0.020). Most MRI scans (74.7%) were performed during remission; 77.9% of MRI scans in MS patients and 68.8% in NMOSD patients were taken during remission (*P* = 0.106). In MRI scans of NMOSD patients, 19.3% (N = 21/109) had normal findings, and 80.7% (N = 88/109) had abnormal findings. Based on the previous classification, 45.9% (N = 50/109) of MRI scans showed NMOSD-specific brain lesions, such as longitudinal corticospinal tracts lesions (10.1%, N = 11/109), extensive hemispheric lesions (14.7%, N = 16/109), periependymal lesions (38.5%, N = 42/109), and cervicomedullary lesions (5.5%, N = 6/109)^15,16^. NMOSD-specific brain lesions were observed in only 38.7% (N = 29/75) of MRI scans when we included only those MRI scans obtained during remission. Of the 199 MRI scans from the patients with MS, 164 (164/199 scans, 82.4%) scans met the Barkhof criteria, and 38 (38/198 scans, 19.2%; one MRI was excluded as it was taken without enhancement) scans showed T1 enhancing lesions; 25.0% (11/44) of MRI scans taken during the acute relapse phase showed T1 enhancing lesions which were identified in 17.5% (27/154) of the MRI scans taken during periods of remission.

### Classification results

We trained a ResNet-18 model that can take 5 channels as input for 25 epochs. We created a 5-channel 2D image by concatenating the selected five axial slices, which we used as input data. Group K-fold is a K-fold validation method that prevents multiple images of one patient from being included in some training set and other images from being included in the validation or test dataset. Batch size was 10, loss function was optimized using the Adam optimizer, and the learning rate was set to 5e−4. The weighted CrossEntropyLoss function was applied to solve the imbalance of the images used. We only used augmented data during model training, not for validation or testing. The test set was used to evaluate the ultimate performance of the model. To minimize the influence on the model's assessment of cases where follow-up scans might not show any major differences or may greatly resemble the baseline scans, the test set consisted only of baseline images of patients without any follow-up images. From the pool of MRI scans of 34 MS patients and 46 NMOSD patients, all of whom had not undergone any follow-up scans, we randomly selected 15 MRI images from each group. Using a different random seed each time, we repeated this process 100 times. The classification results are presented in Table 2. The accuracy of this model in differentiating between NMOSD and MS was 76.1% (95% CI 74.8–77.4) with a sensitivity of 77.3% (95% CI 74.4–80.3) and a specificity of 74.8% (95% CI 72.1–77.5). Positive predictive value (PPV) and negative predictive value (NPV) were 76.9% (95% CI 75.2–78.7) and 78.6% (95% CI 76.7–80.6), with an area under the receiver operating characteristic (ROC) curve of 0.85 (95% CI 0.84–0.86).

**Table 2:** Classification results of multiple sclerosis and neuromyelitis optica spectrum disorder using the proposed architecture.

	Accuracy, %	Sensitivity, %	Specificity, %	PPV	NPV	AUC
Model^a^	76.1 (74.8–77.4)	77.3 (74.4–80.3)	74.8 (72.1–77.5)	76.9 (75.2–78.7)	78.6 (76.7–80.6)	0.85 (0.84–0.86)

PPV, positive predictive value; NPV, negative predictive value; AUC, area under the curve.

^a^Data have been calculated with 100 times experiments and presented with mean and 95% confidence interval.

### Gradient-weighted class activation map (Grad-CAM)

**Figure 1:**
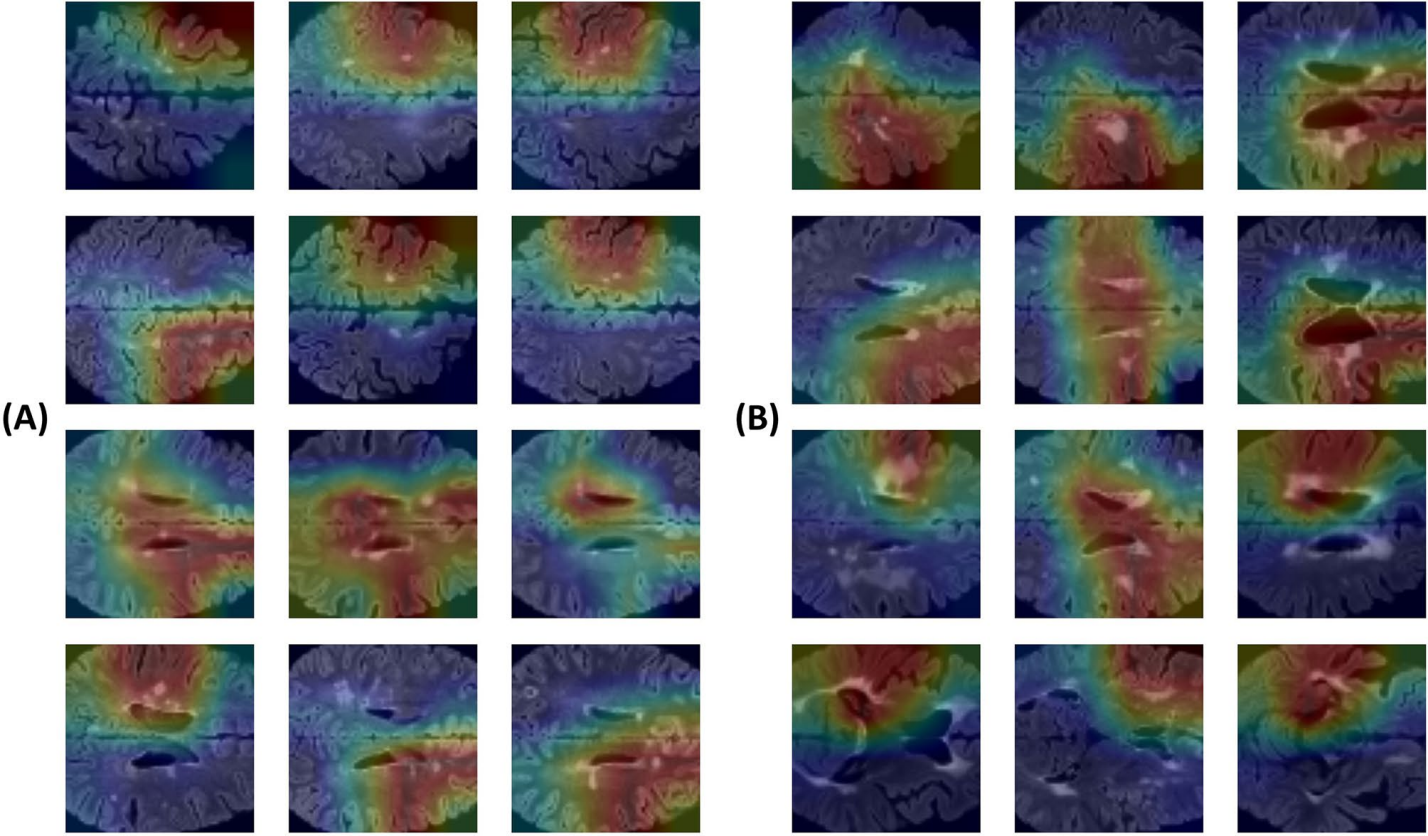
Application of Grad-CAM. (**A**) Grad-CAM results for MS input data, and (**B**) Grad-CAM results for NMOSD. The areas marked in red includes white matter lesions in MS and NMOSD. Grad-CAM, Gradient-weighted Class Activation Map; MS, Multiple Sclerosis; NMOSD, Neuromyelitis Optica Spectrum Disorder.

We generated a gradient-weighted class activation map (Grad-CAM) to evaluate if the five 2D slices selected from the 3D fluid-attenuated inversion-recovery (FLAIR) images represented lesions that could be used to distinguish between MS and NMOSD. Grad-CAM results are shown in Fig. 1. Areas with white matter lesions are highlighted in red, indicating that our ResNet-18 model generated results by recognizing MS and NMOSD lesions in the images during the classification task.

## Discussion

We developed a compact deep learning model with good accuracy and prediction using five axial slices of FLAIR brain MRI for differentiating MS and NMOSD. Further exploration of this model using Grad-CAM showed that white matter lesions were what the model focused on for classification.

Diagnosis of MS can be challenging if patients have atypical clinical presentations. Misdiagnosis of MS could cause patients to undergo hazardous treatment; MS therapies, including interferon beta or fingolimod, can exacerbate NMOSD^17,18^. Serologic testing, which is the major diagnostic criterion for NMOSD, can help differentiate MS from NMOSD, but there are still limitations in the availability of antibody testing, and seronegative cases exist. Misdiagnosis of MS is common, and revision of the McDonald criteria in 2017 raised concerns about misdiagnosis and emphasized the need for systematic identification of typical MRI features, but exclusion of alternative diagnoses is not standardized^1,11,19,20^. Characteristics of brain MRI lesions have also been studied to differentiate between MS and NMOSD. However, we showed that 10% of brain MRIs of patients with the onset of NMOSD met the MS MRI criteria, suggesting it may be challenging to distinguish NMOSD from MS based only on brain MRI at onset^15^. Previously, brain lesions characteristic of NMOSD were observed in 69% of patients with NMOSD during the disease course^16^. Other cross-sectional studies showed lower frequencies of NMOSD-specific brain lesions: 50.9% in chronic phase European patients and 17.7% in chronic phase Chinese patients^21,22^. This indicates that lesions characteristic of NMOSD could be missed outside the acute phase and that different ethnic population, selection bias, or expert knowledge could affect accurate differentiation of the two disorders^6,15^. In our study, only 38.7% of MRI scans performed in the chronic phase showed NMOSD-specific brain lesions, suggesting that it might be challenging to distinguish NMOSD based on MRI in our study population.

Machine learning is an alternative approach to differentiating between NMOSD and MS. Efforts have been made to apply machine learning algorithms to differentiate between MS and NMOSD. Multiple modalities, including functional MRI, white matter lesions, gray matter measures, diffusion tensor imaging, cortical thickness, and cognitive/clinical assessment, were used; a high accuracy of 74% to 84% was attained depending on modality, which can improve our understanding of the characteristics of the disease related to the modalities^13,23^. However, the models used were not fully automated and requiring expert evaluation and selection of the features.

Deep learning models can overcome these obstacles. Only two studies have applied deep learning-based methods to distinguish MS from NMOSD. One study reported 81.3% accuracy of differentiation between MS and NMOSD using hierarchical multimodal fusion models that integrated FLAIR and diffusion tensor imaging (DTI) sequences^24^; the other showed 71.1% accuracy using CNN integrated brain MRI and clinical data^14^. Our deep learning model used only five axial slices of FLAIR MRI data, and showed comparable accuracy (76.1%) with good sensitivity and specificity (77.3%, and 74.8%, respectively).

The deep learning model we used is the residual neural network (ResNet), which is a neural network widely used in the medical field^25–28^. ResNet architecture solves the issue of a relatively limited training dataset and enhances image classification performance by expanding the network's depth. Skip connections are used by ResNet to alleviate degradation issues. Empirical evidence suggests that the training cost of 3D Convolutional neural networks (CNNs) is significantly greater than that of 2D CNNs as more trainable parameters necessitate longer training durations and more training data. The limited dataset size used in this study may potentially have impacted the function of 3D CNNs. Consequently, we employed ResNet 2D CNNs in our study. Data augmentation is a technique for expanding the amount and quality of training datasets to improve the performance of deep learning models. Additionally, data augmentation involves adding missing data points to the initial training data^29^. Using this approach, we attempted to minimize potential issues associated with using the limited amount of FLAIR MRI data. This approach has been used in other studies; a classification task was performed with ResNet and data augmentation with flipping was found to enhance accuracy^30,31^, while a noisy augmented dataset offered superior classification accuracy on ResNet compared to the original dataset^32^.

The complexity of the learning process makes it challenging to interpret deep learning models^33^. Grad-CAM method can provide insight into how deep learning models classify images by facilitating localization of features that the deep learning model focuses on using a heatmap^33,34^; a deep learning model may distinguish between images in ways that are distinct from how humans do^35^. In this study, Grad-CAM revealed that the model focused on white matter lesions to differentiate between MS and NMOSD (Fig. 1). Unknown features of two diseases other than white matter lesions were not recognized with Grad-CAM; white matter lesions therefore appear to be an appropriate area for classification. Further deep learning models with large scale image data from MS and NMOSD could help discover new imaging characteristics.

This study has several limitations. First, this study was conducted with a relatively small number of MRIs in a single center without external validation, which limits generalization of our findings. Second, our model was trained for binary classification, and brain MRIs of healthy subjects were not included in this study. This could be a significant barrier when implementing this model in clinical settings. Third, the clinical state of the disease when MRI scans were performed was not controlled; 68.8% of MRI scans were taken in a chronic remission state. However, given that it may be more challenging to differentiate NMOSD in the chronic phase using MRI data than NMOSD in the acute phase, our findings suggest that this model is useful. Further investigations with extensive data are required to develop a fully automated deep learning model for the diagnosis of CNS demyelinating diseases.

In conclusion, we developed a compact deep learning model based on FLAIR brain MRI data with the ability to differentiate MS from NMOSD. We showed that this model, using the Grad-CAM approach, differentiated between MS and NMOSD based on white matter lesions. This compact deep learning model may aid in the differential diagnosis of MS from NMOSD in clinical practice.

## Methods

### Patients

We prospectively evaluated patients who visited the neurology outpatient clinic of Samsung Medical Center (Seoul, Korea) between May 2016 and May 2020. Patients were enrolled if they had MS or NMOSD, and their diagnosis was performed by two experienced neurologists according to the 2017 McDonald criteria or the international consensus diagnostic criteria for NMOSD, respectively^1,2^. We collected brain MRIs during clinical follow-up; standardized T2-weighted, three-dimensional T1-weighted turbo field echo, and three-dimensional fluid-attenuated inversion recovery images were acquired using a 3.0-T MRI scanner (Philips 3.0 T Achieva, Philips Healthcare, Andover, MA, USA) as described previously^36^. Patients were excluded from the study if (a) AQP4 and MOG antibodies were not assessed, (b) they declined to participate in the study, and (c) they had a history of brain surgery or medical disorders, including cerebral infarction, intracranial hemorrhage, brain tumor or head trauma as these can alter brain MRI findings. We also collected demographic characteristics of the enrolled patients, including gender, age, and seropositivity for AQP4 and MOG antibodies.

The study and all experimental protocols were approved by the institutional review board (IRB) of the Samsung Medical Center; all participants provided written informed consent prior to the commencement of the study, and all methods were performed in accordance with the relevant guidelines and regulations.

### Image preprocessing

Preprocessing is a set of operations performed on an image to improve its quality and make statistical analysis more repeatable and comparable. Image registration is a critical step in various biomedical imaging applications. It provides the ability to align one image with another geometrically and is a prerequisite for all imaging applications that compare datasets across subjects, imaging modalities, or time^37^. We registered FLAIR images to T1 images. This was done using FMRIB (Functional Magnetic Resonance Imaging of the Brain)'s Linear Image Registration Tool (FLIRT). The overall geometry of the brain is unlikely to be altered for scans from the same individual, but each scan may have experienced a translation and/or rotation in space. We employed rigid-body transformation with 12 degrees of freedom to correct for this. We used Freesurfer 6.0 to resample the FLAIR image to 256 size and correct intensity non-uniformity^38^. T1 images were converted to Montreal Neurological Institute (MNI) standard space using FMRIB's Nonlinear Image Registration Tool (FNIRT), and coefficient maps were obtained in this process. FLAIR images were converted to MNI standard space using FSL's applywarp function, which applies the FNIRT's coefficient map to other images. To obtain only the brain part without the background, we cropped the FLAIR image to 128 size.

**Figure 2:**
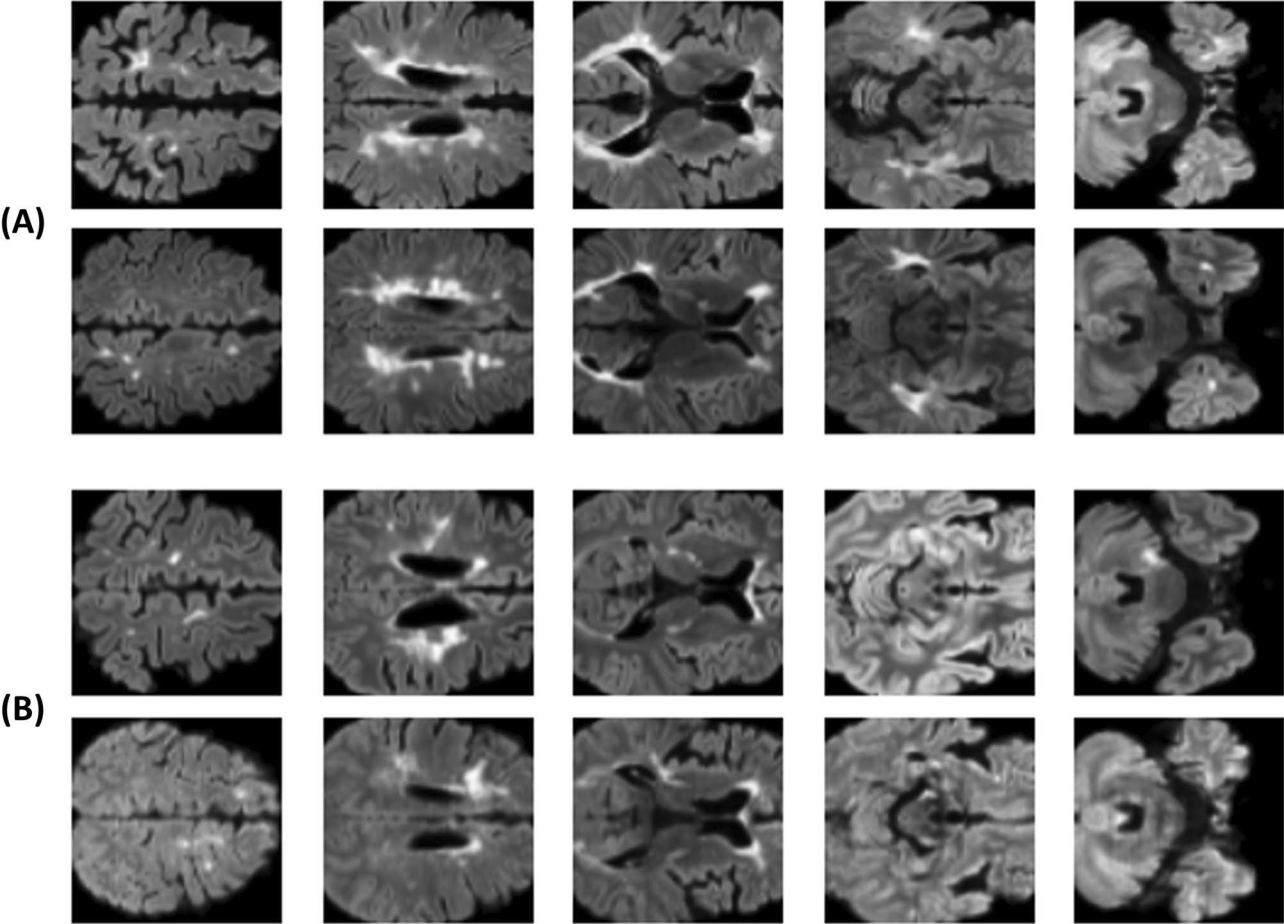
Example of 2D FLAIR image input data for use in the classification model. (**A**) Images from two patients with multiple sclerosis, (**B**) images from two patients with neuromyelitis optica spectrum disorder. One slice is one channel, so the five images in a row were merged into one input image with five channels.

A total of five axial slices were chosen at 20 slice intervals before and after to distinguish multiple sclerosis from NMOSD based on the position of the lateral ventricle where lesions are present in both disorders but the morphology of the lesions differs (Fig. 2)^10^. The five axial slice positions were the most similar positions presented on the report of Matthews and colleagues^9^, representing the cortical area, deep white matter area, lateral ventricle, basal ganglia, and brainstem/cerebellum. We replaced one slice with one channel, resulting in a five-channel input image.

### Convolutional neural networks

CNNs is a deep learning method that trains several layers. It is used for a variety of computer vision applications and is very efficient^39–41^. In general, a CNN consists of three main neural layers: convolutional layers, pooling layers, and fully connected layers. Convolutional layers are at the core of a CNN. Convolution is a linear process that, like a conventional neural network, multiplies a set of weights with the input in the context of a convolutional neural network. Multiplication is done between an input array and a two-dimensional array of weights, known as a filter or a kernel, because the approach was designed for two-dimensional input data. A single value is produced by multiplying the filter by the input array once. A two-dimensional array of output values representing an input filter is produced when the filter is applied to the input array more than once. The two-dimensional output array from this operation is known as a feature map. Once a feature map has been generated, each value is passed through a nonlinearity. The function of the pooling layer is to reduce the dimensions by pooling feature maps. It also collects and enhances the features of the extracted image. A fully connected layer is used in a classification task, and a likelihood function is used to calculate the likelihood probability of each image class from the fully connected layer. The most probable labels serve as classifiers throughout the CNN and are output as classification results.

### Data augmentation

High-quality, abundant data is critical in the development of deep learning models. A deficit of training data can lead to overfitting^42^. The classification problem addressed in this paper lacks sufficient data to provide a deep learning architecture. Therefore, we performed data augmentation based on the training set using the following two methods to achieve the desired accuracy. The first data augmentation method we used was the RandomHorizontalFlip. RandomHorizontalFlip is a type of image data augmentation that flips the input image horizontally with a given probability. The second data augmentation method we used is RandomNoise^43^. RandomNoise is a simple form of data augmentation that adds noise sampled from a normal, random distribution. By training a neural network on noisy data, robust neural networks that proficiently generalize, even on noisy images, can be generated.

### Model architecture

**Figure 3:**
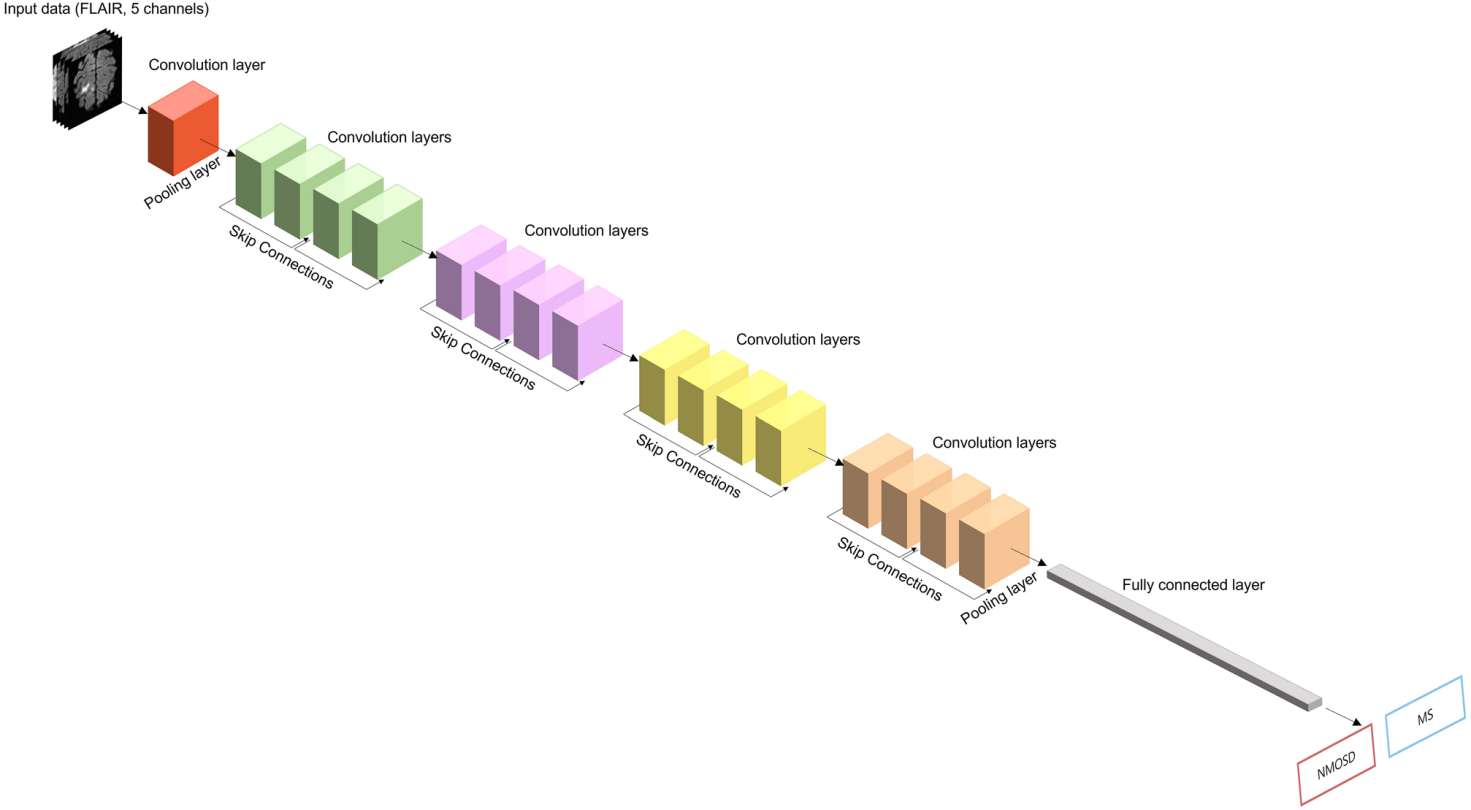
The architecture used to distinguish neuromyelitis optica spectrum disorder from multiple sclerosis. This architecture is based on a residual neural network. The output class of ResNet-18 was changed to 2 and the input data was expanded to 5 channels.

We used a model based on the ResNet CNN model^44^. There are several types of ResNet, such as ResNet-18, ResNet-50, and ResNet-101. In ResNet-n, n is the number of layers in the network, and as n increases, the number of computations increases, as well as the performance of the network. We used ResNet-18 with some changes; ResNet18 is a CNN model with a 72-layer architecture and 18 deep layers. ResNet18 consists of one 7 × 7 convolutional layer, two pool layers, eight residual units, and one fully connected layer. Each of the residual units contains two 3 × 3 convolutional layers. Here, we changed the input image of ResNet-18 to five channels and the output class to two types. Figure 3 shows the modified ResNet architecture used in this study to differentiate between MS and NMOSD.

### Gradient-weighted class activation map (Grad-CAM)

Grad-CAM is a generalization of the class activation map (CAM) that finds weights through gradients as follows^34,45^:

$${\alpha }_{k}^{c}= \frac{1}{z}\sum_{i}\sum_{j}\frac{\partial {y}^{C}}{\partial {A}_{ij}^{k}}$$

In the final convolutional layer, we allowed the gradients of any target concept score (logits for any class of interest) to flow. Specific aspects in the image for predicting that concept could then be highlighted on a coarse localization map by computing a significance score based on the gradients. To express this more technically, we computed the gradient of the class C logits concerning the activation maps of the final convolutional layer. Then we averaged the gradients over each feature map to determine a significance score as expressed below:

$${L}_{\mathrm{Grad}-\mathrm{CAM}}^{c}= ReLU\left(\sum_{k}{\alpha }_{k}^{c}{A}^{k}\right)$$

where c is the class of interest, k is the index of the activation map in the final convolutional layer, ${y}^{c}$ is the score for class c before softmax, and ${A}^{k}$ is the feature map of the k-th channel of the last CNN layer. The alpha value indicates the significance of feature map k for the target class c. The values are then added together after multiplying each activation map by its significance score. ReLU nonlinearity is also used in the summation to take into account only those pixels that positively affect the score of the class of interest.

### Statistical analysis

Clinical characteristics of the enrolled patients are presented with appropriate summary statistics. Continuous data are shown as means with standard deviations or medians with inter-quartile ranges (IQRs). Categorical variables are presented as absolute and relative frequencies. We compared demographic findings between the two groups (MS versus NMOSD) using the Chi-square test or Fisher’s exact test for categorical variables. Student’s t-tests or Mann–Whitney U tests were used to compare continuous variables. The performance of our model was evaluated using appropriate classification metrics, namely accuracy, sensitivity, specificity, PPV, NPV, and area under the ROC curve. The results of 100 experiments are presented with means and 95% confidence intervals (CIs). All statistical analyses were performed using SPSS for Windows version 20.0 (IBM, Armonk, NY, USA) or R software version 4.2.1. Statistical significance was defined as a two-tailed *p*-value < 0.05.

## Data Availability

The datasets used and/or analyzed during the current study can be available from the corresponding author on reasonable request.
